# Supplementary figures and images for: Abundance of arthropods as food for meadow bird chicks in response to short- and long-term soil wetting in Dutch dairy grasslands
Source: PeerJ. 2019 Sep 10;7:e7401. doi: 10.7717/peerj.7401 (PMC6743474; doi:10.7717/peerj.7401)

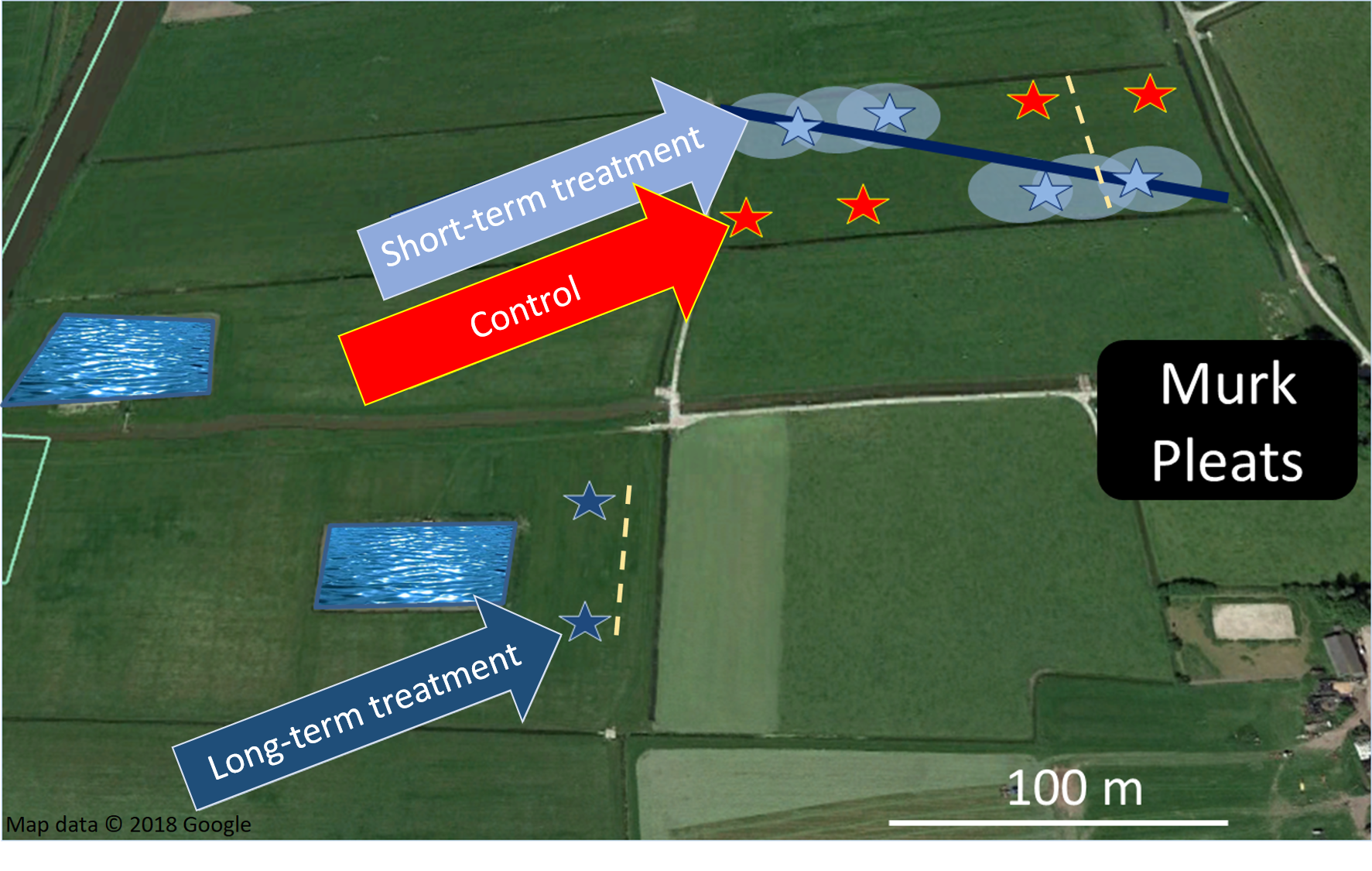

Supplement: Supplemental Information 1 — On the right Murk Pleats indicates the location of the farmer’s house. The stars are the sites where the replicates were placed and the blue line with the light blue circles represents the sprinkler system. © Google Earth 7.3.2.5776 image taken Jun 1 2017. [file peerj-07-7401-s001.png]

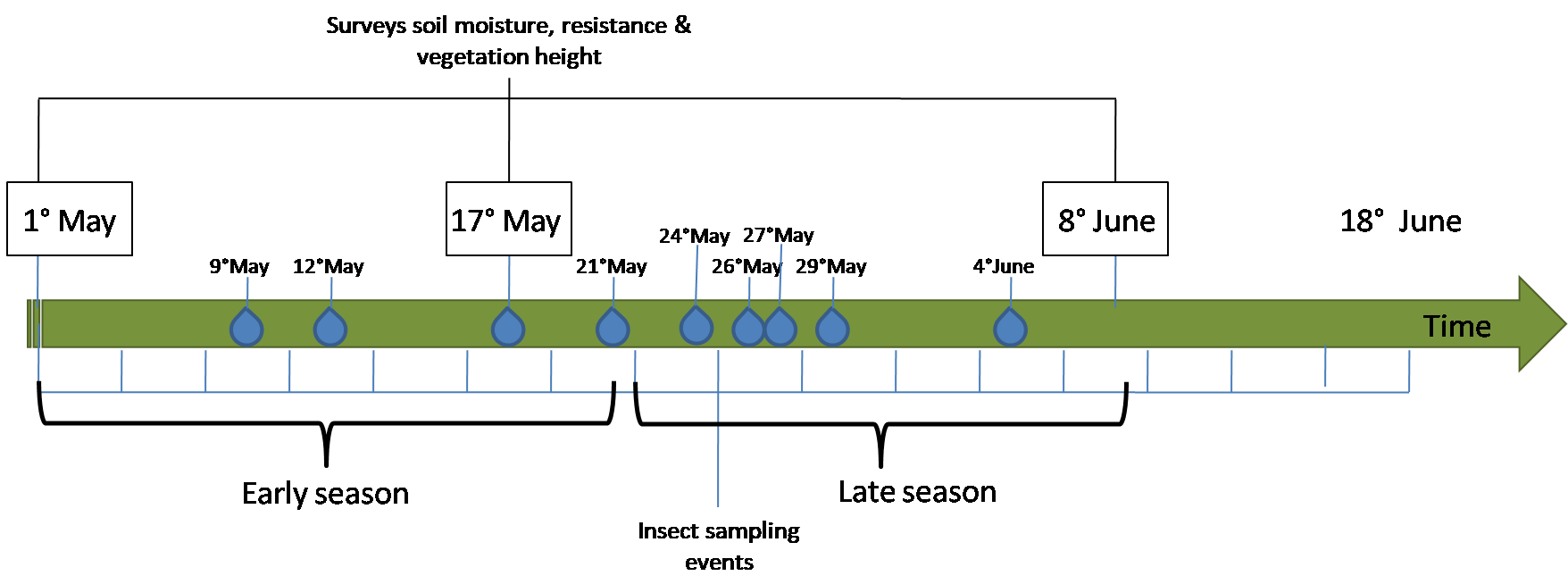

Supplement: Supplemental Information 2 — The blue drops represent the irrigation events. [file peerj-07-7401-s002.png]
