# Supplementary material for: Abundance of arthropods as food for meadow bird chicks in response to short- and long-term soil wetting in Dutch dairy grasslands
Source: PeerJ. 2019 Sep 10;7:e7401. doi: 10.7717/peerj.7401 (PMC6743474; doi:10.7717/peerj.7401)
Supplement: Supplemental Information 3 [file peerj-07-7401-s003.docx]

| **Plant species** | **Control/ Irrigated** | **Relative  abundance** | **Near water** | **Relative abundance** |
| --- | --- | --- | --- | --- |
| *Alopecurs geniculatus* | 0 | 0.00 | 2 | 0.01 |
| *Alopecurus pratensis* | 13 | 0.09 | 1 | 0.01 |
| *Bellis perennis* | 3 | 0.02 | 2 | 0.01 |
| *Bromus hordaceus* | 13 | 0.09 | 1 | 0.01 |
| *Cardamine pratensis* | 3 | 0.02 | 3 | 0.02 |
| *Cerastium fontanum vulgare* | 8 | 0.05 | 0 | 0.00 |
| *Dactylis glomerata* | 35 | 0.23 | 22 | 0.15 |
| *Elytrigia repens* | 10 | 0.07 | 4 | 0.03 |
| *Festuca rubra* | 0 | 0.00 | 7 | 0.05 |
| *Holcus lanatus* | 2 | 0.01 | 1 | 0.01 |
| *Lolium perenne* | 23 | 0.15 | 29 | 0.19 |
| *Poa pratensis* | 0 | 0.00 | 1 | 0.01 |
| *Poa trivialis* | 1 | 0.01 | 11 | 0.07 |
| *Ranunculus acris* | 11 | 0.07 | 5 | 0.03 |
| *Ranunculus ficaria* | 1 | 0.01 | 2 | 0.01 |
| *Ranunculus repens* | 1 | 0.01 | 0 | 0.00 |
| *Rumex acetosa* | 7 | 0.05 | 13 | 0.09 |
| *Sonchus arvensis* | 1 | 0.01 | 0 | 0.00 |
| *Sonchus asper* | 1 | 0.01 | 0 | 0.00 |
| *Taraxacum officinale* | 11 | 0.07 | 25 | 0.17 |
| *Trifolium pratense* | 3 | 0.02 | 18 | 0.12 |
| *Trifolium repens* | 3 | 0.02 | 3 | 0.02 |
| **Total** | **150** |  | **150** |  |
| **Species richness** | **19** |  | **18** |  |
| **Diversity (H')** | **-0.09** |  | **-0.09** |  |
