# Supplementary material for: Abundance of arthropods as food for meadow bird chicks in response to short- and long-term soil wetting in Dutch dairy grasslands
Source: PeerJ. 2019 Sep 10;7:e7401. doi: 10.7717/peerj.7401 (PMC6743474; doi:10.7717/peerj.7401)
Supplement: Supplemental Information 4 — The equation (weight) = B0 ( length )^B1 was used to calculate the biomass (Roger et al., 1978). The table shows the numbers of specimens per Order in each type of trap, the coefficients for the equation and the correspondent biomass (mg). [file peerj-07-7401-s004.docx]

| **Order** | **Life stage** | **Sticky traps** | **Pitfall traps** | **B_0_ ± SE** | **B_1_ ± SE** | **Biomass (mg)** |
| --- | --- | --- | --- | --- | --- | --- |
| Coleoptera | Adult  Larvae | 521  - | 498  9 | -3.460±0.105  -0.792±0.652 | 2.790±0.050  0.571±0.055 | 1537.14 |
| Diptera | Adult  Larvae | 369564  - | 38793  1 | -3.293±0.115  -3.731 ±0.472 | 2.366±0.078  0.356±0.051 | 36725.75 |
| Lepidoptera | Adult  Larvae | 508  - | 346  3 | -4.037±0.133  -5.137±0.319 | 2.903±0.080  2.809±0.146 | 5472.28 |
| Aranaea |  | 68 | 2332 | -3.106±0.189 | 2.929±0.294 | 37.72 |
| Hemiptera |  | 2313 | 51 | -2.998±0.113 | 2.270±0.081 | 1108.95 |
| Hymenoptera | | 4682 | 1373 | -3.871±0.108 | 2.407+0.060 | 822.26 |
| Acarina |  | 192 | 6495 | +3.682±0.171 | 2.761 ±0.309 | 6.18 |
| Collembola* |  | 24 | 67 | - | - | - |
| Chilopoda* |  | - | 1 | - | - | - |
| Embioptera* |  | 1 | 4 | - | - | - |
| Isopoda* |  | - | 1 | - | - | - |
| Megaloptera* |  | 2 | 1 | - | - | - |
| Neuroptera* |  | 7 | 2 | - | - | - |
| Mollusca* |  | - | 80 | - | - | - |
| Odonata* |  | 3 | - | - | - | - |
| Orthoptera* |  | 1 | - | - | - | - |

For Orders marked with * there was no biomass conversion coefficient available in Rogers et al., 1977, however, the number of specimens found in the sample was very low and we do not expect them to be relevant to the analysis.
